# 4287 Extracellular vesicles as biomarkers for early detection of pancreatic cancer

**DOI:** 10.1017/cts.2020.67

**Published:** 2020-07-29

**Authors:** Charles P Hinzman, Shivani Bansal, Yaoxiang Li, Partha Banerjee, Amrita Cheema

**Affiliations:** 1 Georgetown - Howard Universities; 2 Georgetown University Medical Center

## Abstract

OBJECTIVES/GOALS: Pancreatic ductal adenocarcinoma (PDAC) is projected to become the second leading cause of cancer-related deaths by 2030. Though many other cancers have seen improvements in patient survival rates, patients diagnosed with PDAC have a 5-year survival rate of only ~9%. A major contributor to decreased survival is late-stage diagnosis of the disease. New methods of early detection are urgently needed. Extracellular vesicles (EVs) are secreted from cells of all tissue types into the circulation. EVs play important roles in a variety of diseases. They have shown to promote cancer progression and they are being studied as potential biomarkers for disease diagnosis. The purpose of this study was to perform qualitative and quantitative characterization of small-molecule profiles of EVs derived from various pancreatic cancer (PC) and normal pancreas cell lines, to provide proof-of-concept for evaluating the efficacy of leveraging EVs as potential biomarkers of PDAC. METHODS/STUDY POPULATION: EVs were isolated from the conditioned media of six PC and two normal pancreas cell lines using differential ultracentrifugation with filtration. EV enrichment was validated using quantitative ELISA, immunoblot and transmission electron microscopy. Targeted liquid chromatography coupled to mass spectrometry (LC-MS/MS) and untargeted (UPLC-QTOF-MS) metabolomics were used to analyze the biochemical composition of EVs. RESULTS/ANTICIPATED RESULTS: The biochemical profile of PC EVs was found to be significantly different from the profiles of normal cell EVs. Interestingly, amino acids were downregulated in PC EVs as compared to normal cell EVs. However, PC EVs were enriched in lactate and malate. PC EVs also had significant upregulation in other small molecules such as xanthosine, guanosine diphosphate and nicotinamide. DISCUSSION/SIGNIFICANCE OF IMPACT: Our results indicate that the biochemical characterization of EVs using metabolomics has the potential to yield biomarkers which can delineate cancer cell-derived EVs from normal cell-derived EVs. Further work will test the clinical significance of these findings by similar analyses of plasma of PDAC patients. Furthermore, these profiles may be detectable before progression of the disease to late-stage PDAC, leading to the development of assays for earlier diagnosis in patients.

